# Enhancing the pricing efficiency of financial assets with an optimized bayesian network based on efficient fusion

**DOI:** 10.1371/journal.pone.0347047

**Published:** 2026-05-08

**Authors:** Qi Fu, Xiaotong Li

**Affiliations:** 1 School of Finance, Shanghai Lixin University of Accounting and Finance, Shanghai, China; 2 Faculty of Business Economics, Shanghai Business School, Shanghai, China; University of Salerno: Universita degli Studi di Salerno, ITALY

## Abstract

To address the limitations of traditional pricing models regarding accuracy and adaptability in high-frequency trading, this study presents a Transformer-based Efficiently-Fused Optimized Bayesian Network (Trans-EFOBN) for financial asset pricing. The framework integrates a masked transformer with temporal logic constraints to extract sequential features and combines a Dynamic Bayesian Network (DBN) to establish hierarchical structural dependencies between macro factors and micro market variables. This design does not aim to establish strict econometric causality but instead leverages an end-to-end learning mechanism to simultaneously optimize feature representation and network parameters. Empirical analyses utilizing minute-level high-frequency data of the CSI 300 constituent stocks from 2019 to 2024 in the Wind database demonstrate substantial performance gains: the mean absolute error (MAE) decreases to 0.037 (approximately 25% lower than the baseline static Bayesian model), while R² attains 0.86. In simulated trading scenarios incorporating transaction costs and slippage, the proposed model yields an annualized return of 14.2% and a Sharpe ratio of 0.95. The results indicate that integrating structural dependency logic with dynamic probabilistic inference significantly enhances asset pricing efficiency and interpretability, providing robust technical support for high-frequency quantitative trading.

## Introduction

In evolving financial market, trading strategies in high-frequency trading scenarios must achieve extreme precision while responding instantaneously to market dynamics [1,2]. This requirement poses unprecedented challenges for the timeliness and accuracy of financial asset pricing. With the integration of financial technology and data intelligence, quantitative trading has increasingly relied on multi-source heterogeneous data to capture changes in market microstructures and to uncover potential arbitrage opportunities [3]. Such developments challenge traditional pricing models grounded in economic theory or statistical assumptions. Traditional models frequently assumed a static market environment and linear structural dependencies, which limited their effectiveness under dynamic and complex market conditions. In high-frequency trading, where data exhibited strong nonlinearity and instability, these models often failed to meet the requirements of precision, responsiveness, and robustness.

In recent years, the adoption of artificial intelligence in finance introduced deep neural networks, graph neural networks (GNN), and causal inference frameworks into asset pricing research [4], aiming to overcome the limitations of traditional approaches. However, substantial gaps remained. Mainstream deep learning models, such as Long Short-Term Memory (LSTM) networks, captured short- and long-term temporal dependencies effectively [5] but lacked explicit causal structures, obscuring economic transmission mechanisms underlying price fluctuations. In contrast, probabilistic graphical models, such as Bayesian networks, provided strong interpretability and causal reasoning [6,7], yet their structural learning efficiency was limited when applied to high-dimensional, high-frequency, nonlinear financial data. Current empirical studies frequently struggle to balance predictive precision with the transparency of model structures. Rather than pursuing strict econometric causal inference, this study utilizes a hybrid architecture to maintain high-frequency performance while leveraging structured probabilistic representations for enhanced logical consistency.

The Transformer-based Efficiently-Fused Optimized Bayesian Network (Trans-EFOBN) framework proposed in this study ensures the prediction process adheres to temporal sequential logic through a masking mechanism. This integrated model enables the system to capture nonlinear market sentiment while mapping structured pathways of risk transmission. Compared with existing studies, the contributions of this study are threefold:

- Unlike single predictive models, the proposed Trans-EFOBN framework integrates the long-range temporal feature extraction of Transformers with the logical inference advantages of hierarchical Bayesian networks. This complementary design demonstrates the necessity of hybrid architectures in high-frequency financial scenarios, enabling a transition from association modeling to structural dependency modeling while maintaining high precision.
- Within the Transformer module, a temporally causal masking mechanism is implemented to ensure that all predictions are strictly derived from historical information. Simultaneously, attention weights dynamically guide the evolution of the Bayesian network structure, effectively resolving structural update lag and capturing real-time fluctuations in dependency intensity.
- An end-to-end joint optimization mechanism utilizes a composite loss function that combines prediction error, directed acyclic graph (DAG) constraints, and a risk-adjusted term. This substantially improves the model’s robustness during periods of high market volatility.

**Fig 1:**
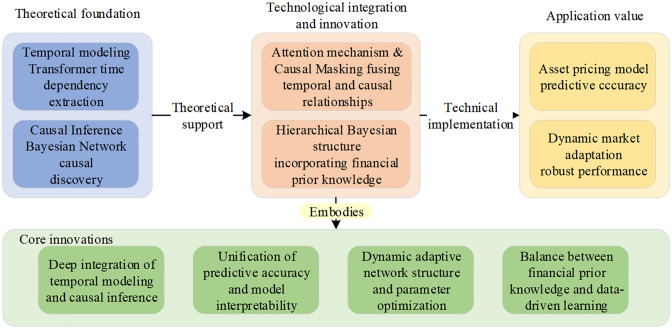
Research framework.

By selecting highly liquid constituent stocks from the CSI 300 index as a representative case, this study aims to verify the applicability and pricing efficiency of the hybrid framework within complex and volatile market environments. The overall research framework is illustrated in Fig 1.

## Recent related work

### Research status of financial asset pricing

Financial asset pricing remains a cornerstone of financial engineering and quantitative economics. Seong and Nam (2022) [8] developed a complex financial network model to forecast fluctuations in global financial indices. The authors revealed that asset prices were significantly influenced by network structures and introduced a systemic linkage perspective to asset pricing. Barro et al. (2022) [9] proposed a stochastic programming model for dynamic portfolios incorporating derivatives, which effectively captured the risk–return structure of assets and enriched the theoretical foundation for dynamic hedging strategies in asset pricing. Harb et al. (2023) [10] examined corporate accounting manipulation before and after financial engineering practices using Benford’s Law, highlighting the critical role of information quality in market efficiency and asset pricing. Keshari and Gautam (2023) [11] employed bibliometric methods to trace the evolution of global asset pricing research, identifying the emergence and convergence of research paradigms such as factor models and behavioral finance. Li et al. (2023) [12] examined how digital finance alleviated corporate financing constraints, thereby indirectly promoting more accurate asset valuation and emphasizing the role of technological empowerment in capital allocation. Peng et al. (2024) [13] conducted an empirical study on the impact of governmental digital attention on corporate digital transformation and financing constraints. Their analysis suggested that policy interventions could indirectly enhance the efficiency of asset pricing by improving information transparency.

Moreover, recent studies have expanded both macro-level perspectives and micro-level complexity of financial asset pricing. Batrancea et al. (2023) [14] empirically examined how infrastructure shaped economic growth, highlighting the fundamental role of macroeconomic conditions in long-term asset valuation. To address the nonlinear and dynamically interactive nature of financial markets, Akgüller et al. (2025) [15,16] applied Fractional Transfer Entropy Networks and delayed fractional-order models. They uncovered long-memory characteristics in global equity markets and revealed how external shocks, such as seismic disasters, affected specific sectors through complex mechanisms. At the investment decision level, the distributionally robust stochastic optimization approach proposed by Batrancea et al. (2025) [17] further demonstrated the need to enhance model robustness under high uncertainty. In summary, although existing literature expanded the connotations of asset pricing across macro and micro dimensions, significant challenges remained in identifying logically consistent structural paths within massive high-frequency “noise” data. Most cutting-edge studies either focused on capturing long-memory temporal features or emphasized static risk transmission analysis, while the dynamic fusion mechanism between both was rarely discussed. Rather than attempting to reconstruct traditional econometric causal frameworks, this study aims to bridge the deficiencies of pure statistical models in explaining price formation logic by optimizing Bayesian networks. Consequently, this approach provides a more structurally robust pricing perspective within highly uncertain market environments.

### Application status of deep learning in financial modeling

To overcome the limitations of traditional models, literature has evolved along two technical pathways: enhancing Model transparency / Structural logic and optimizing sequence feature extraction. Bayesian networks were among the first to excel in in financial systemic risk modeling due to their explicit causal inference and graphical structures. Zhou et al. (2022) [18], Wei et al. (2023) [19], and Chan et al. (2023) [20] systematically represented the dependency structures among multiple risk factors in shipping services, energy finance, and systemic financial risk, respectively. They utilized sliding-window mechanisms to capture dynamic market changes. Subsequent studies explored the decision-support potential of hybrid architectures. Song et al. (2024) [21] showed that combining LSTM with Bayesian networks could enhance causal modeling of time series. Meng et al. (2024) [22] and Zheng (2024) [23] demonstrated the value of Bayesian structures in modeling probabilistic dependencies and supporting complex decision-making under uncertainty, such as in multi-factor accident attribution and project financing evaluation.

The emergence of the Transformer architecture marked a major breakthrough in sequence modeling. Wang et al. (2022) [24] confirmed that deep Transformers outperformed traditional Recurrent Neural Networks and LSTM networks in capturing long-term temporal dependencies and volatility patterns. Subsequent improvements focused on enhancing adaptability in complex financial environments. Xu et al. (2023) [25] and Wei et al. (2024) [26] improved trend detection and high-dimensional feature extraction through linear-structure optimization and heterogeneous data fusion. Mishra et al. (2024) [27] and Yañez et al. (2024) [28] further incorporated parallel multi-structure designs and frequency-domain decomposition techniques, strengthening the model’s responsiveness to market sensitivity, nonlinear patterns, and cyclical dynamics.

Although models such as Transformer and TCN achieved breakthroughs in time-series forecasting, their predictive processes frequently lacked structural transparency. While increasing model complexity improved short-term goodness of fit, models remained prone to failure during extreme market fluctuations without constraints on logical dependencies between variables. Meanwhile, traditional Bayesian networks possessed advantages in logical inference, yet encountered difficulties in directly processing high-dimensional and non-stationary financial sequences.

### Research gaps and innovations

Table 1 provides a critical comparison of primary asset pricing methodologies. Unlike previous studies that either relied in isolation on deep learning for feature extraction or on Bayesian networks for static inference, this study constructs a dynamic feedback mechanism. Comparative analysis revealed that existing hybrid models frequently ignored the structural characteristics of “asymmetric information transmission” within high-frequency data. The combination of a causal masking mechanism and hierarchical DBN accurately fills this gap, enhancing the ability to capture variable correlation changes during extreme market conditions. Consequently, this approach provides robust support for the uniqueness of the proposed framework within complex financial networks.

**Table 1:** Comparison of related literature with this study.

Reference	Main Method	Research Objective	Innovation
Seong and Nam (2022)	Complex financial network model	Predict global financial index volatility	Introduced a systemic interconnection perspective for asset pricing
Barro et al. (2022)	Stochastic programming model	Dynamic portfolio management and risk hedging	Strengthened the theoretical basis of dynamic hedging strategies
Harb et al. (2023)	Benford’s Law	Examine the impact of accounting manipulation on market efficiency	Emphasized the role of information quality in market efficiency
Keshari and Gautam (2023)	Bibliometric analysis	Trace the evolution of global asset pricing research	Identified trends in factor models and behavioral finance
Li et al. (2023)	Digital finance analysis	Explore how digital finance alleviates corporate financing constraints	Highlighted the role of technology in capital allocation
Peng et al. (2024)	Empirical study	Assess the government digital attention on corporate financing constraints	Policy intervention improves transparency, enhancing pricing efficiency
Zhou et al. (2022)	Bayesian network	Global shipping risk assessment	Systematically characterized causal relationships among risk factors
Wei et al. (2023)	Bayesian network	Energy finance risk warning	Improved risk warning capability and dynamic perception
Chan et al. (2023)	Sliding-window Bayesian network	Systemic financial risk modeling	Captured dynamic dependencies among market variables
Song et al. (2024)	LSTM combined with a Bayesian network	Financial time series and causal relationship modeling	Enhanced causal modeling and model interpretability
Meng et al. (2024)	N–K model combined with a Bayesian network	Transmission pathway analysis of maritime collision incidents in China	Supported multi-factor decision-making for financial insurance valuation
Zheng (2024)	Bayesian network	Project financing risk assessment	Strengthened uncertainty management in investment decisions
Wang et al. (2022)	Deep Transformer model	Stock index prediction	Captured long-term temporal dependencies and volatility patterns
Xu et al. (2023)	Multi-attention and linear Transformer	Financial time series prediction	Improved convergence efficiency and trend detection
Wei et al. (2024)	Transformer fused with multi-source financial data	Risk analysis and high-dimensional modeling	Enhanced extraction of temporal and semantic features from heterogeneous data
Mishra et al. (2024)	Multi-Transformer parallel structure	Market volatility prediction	Improved scalability for risk assessment and market sensitivity analysis
Yañez et al. (2024)	Frequency decomposition combined with the Transformer	Stock index prediction	Improved responsiveness to nonlinear and cyclical patterns
This study	A Transformer combined with a hierarchical Bayesian network	Financial asset pricing	Innovatively fused a Transformer and a Bayesian network, improving interpretability and responsiveness

In summary, existing research exhibited two core deficiencies in asset pricing modeling: deep learning models prioritized predictive accuracy at the cost of black-box risks, and Bayesian networks encountered performance bottlenecks in dynamic multivariate interactions. Addressing these issues, this study innovates in hierarchical modeling and methodological fusion by designing a pricing framework that integrates a causal masking mechanism. The core objective emphasizes utilizing attention-guided structural dependencies to enhance the capture of transmission paths between market sentiment and fundamentals, rather than inferring formal causal effects. This integration improves predictive performance empirically and establishes a logical foundation for the intelligent advancement of high-frequency quantitative trading strategies.

## Methods of applying bayesian networks to financial asset pricing

### Overall framework design of the financial asset pricing model

**Fig 2:**
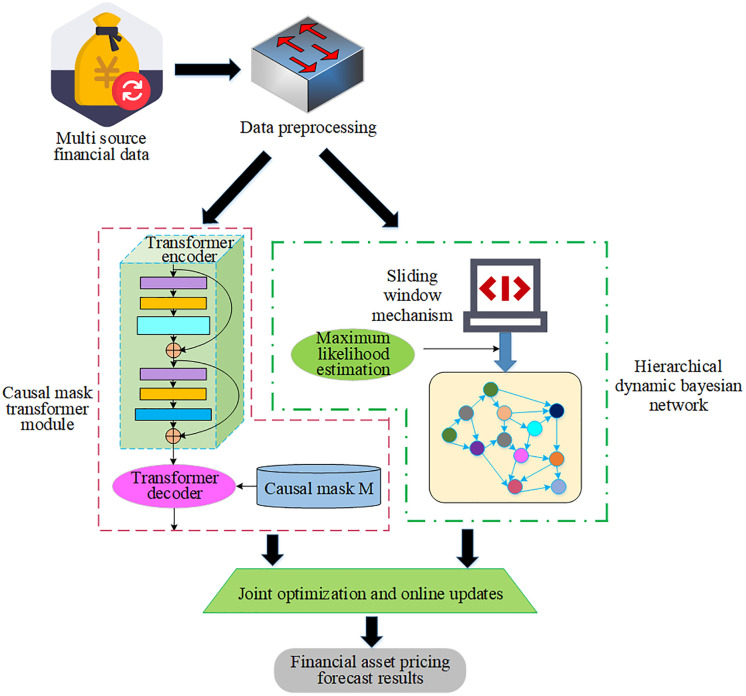
Architecture of the Trans-EFOBN-based financial asset pricing model.

The Trans-EFOBN framework establishes a pricing system that balances predictive performance with logical transparency by integrating a causally masked Transformer with a hierarchical DBN (Fig 2). This framework operates through three synergistic modules: (1) the Transformer module extracts temporal features using a “causal masking” mechanism to ensure strict adherence to temporal unidirectionality; (2) the hierarchical DBN constructor builds structured paths reflecting probabilistic dependencies based on attention weights; (3) the joint optimization module updates feature representations and network parameters simultaneously via an end-to-end differentiable framework. Rather than inferring formal econometric causal effects, this architecture represents the transmission logic of macro shocks to micro stock prices through probabilistic dependency chains. Consequently, the proposed model provides a more interpretable pricing benchmark than pure black-box models within complex high-frequency environments.

### Design and analysis of the transformer module

**Fig 3:**
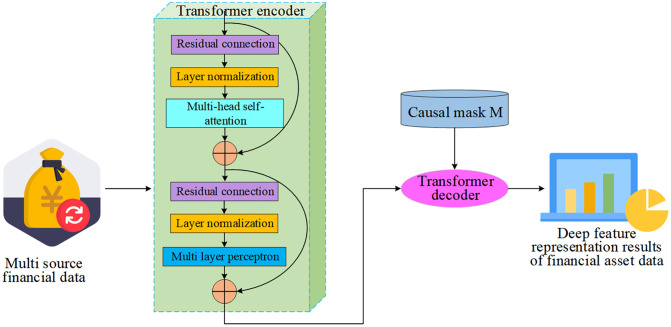
Diagram of the Transformer module with temporally causal masking mechanism.

To accurately extract long-term dependencies and latent causal structures in high-frequency financial time series, this study integrates a Transformer module constrained by a temporally causal masking mechanism into the asset pricing model [29], as illustrated in Fig 3. The primary objective of this module is to transform the input sequences of multi-source financial variables into deep feature representations that adhere to temporal causality, thereby establishing structural and parametric foundations for subsequent Bayesian network modeling.

Let the input sequence over *l* consecutive time steps be denoted as $X_{t-l+1:t}\in R^{l\times d}$, where each time step *t* contains *d* financial variables. The sequence undergoes linear projection and positional encoding *t*o generate feature representations enhanced with temporal information. This procedure constitutes the initial input to the Transformer, as defined in Equation (1):


$$H_{\text{0}}\;=X_{t-l+1:t}\;\cdot W_{in}\;+PE$$ (1)

In Equation (1), $W_{in}$ represents the input projection matrix, and PE denotes the positional encoding matrix used to retain the sequential order of the time steps.

Within each layer of the Transformer with temporally causal masking, a Multi-Head Self-Attention mechanism is employed to model the features at each time step within the sequence. In this mechanism, the query (*Q*), key (*K*), and value (*V*) vectors are derived through linear projections. The attention scores are computed using the scaled dot-product formulation, incorporating a temporally causal masking mechanism *M* to restrict access to future time steps. This computation ensures that pricing predictions adhere to the temporal causality principle, as shown in Equations (2), (3):


$$A^{\left(h\right)}=\text{softmax}\left(\frac{Q^{\left(h\right)}\left(K^{\left(h\right)}\right)T}{\sqrt{d_{k}\;}}\;+M\right)$$ (2)

$$Head^{\left(h\right)}=A^{\left(h\right)}\cdot V^{\left(h\right)}$$ (3)

Here, $A^{\left(h\right)}$ represents the attention scores for the *h*-th attention head, calculated via the scaled dot-product. The matrix $M\in R^{l\times l}$ denotes the temporally causal masking matrix that prevents the model from attending to future time steps, thereby ensuring that only current and past information is used for prediction. The mask matrix $M\in R^{l\times l}$ is defined as in Equation (4):


$$M_{i,j}=\left\{ \begin{array}{cc} @cc@0 & if\text{\textbackslash{}hspace\{0.33em}\}i\geq j\\ -\infty & if\text{\textbackslash{}hspace\{0.33em}\}i<j \end{array}\right.$$ (4)

In Equation (4), $M_{i,j}$ is an element of the causal mask matrix *M*, indicating the temporal relationship between time steps *i* and *j*.

This mechanism effectively prevents information leakage and ensures that the model bases its pricing predictions solely on present and historical data.

The final output of the multi-head attention is formulated in Equation (5):


$$MultiHead\left(H_{l}\;\right)=Concat(Head^{\left(1\right)},\ldots ,Head^{\left(h\right)})\cdot W_{O}$$ (5)

In Equation (5), $Head^{\left(h\right)}$ denotes the output of the *h*-th attention head, obtained by applying the attention scores $A^{\left(h\right)}$ to the value matrix $V^{\left(h\right)}$. $MultiHead\left(H_{l}\;\right)$ represents the final output of the multi-head attention mechanism, computed by concatenating the outputs of all attention heads $Head^{\left(1\right)}$ to $Head^{\left(h\right)}$, followed by a linear transformation with the output projection matrix $W_{O}$.

To bolster stability and nonlinear representation capability, the Transformer framework applies standard residual connections, layer normalization, and feed-forward networks [30]. To support DBN construction, the model extracts the long-term dependency structure by averaging average attention weights across all heads, as shown in Equation (6):


$$\alpha_{ij}\;=\frac{\text{1}}{h}\sum_{k=1}^{h}\frac{\text{1}}{l}\sum_{k=1}^{l}\overset{\left(k\right)}{\underset{t}{A}}\left[i,j\right]$$ (6)

In Equation (6), $\alpha_{ij}$ denotes the aggregate attention strength from feature *j* to feature *i* across the input sequence.

### DBN modeling

**Fig 4:**
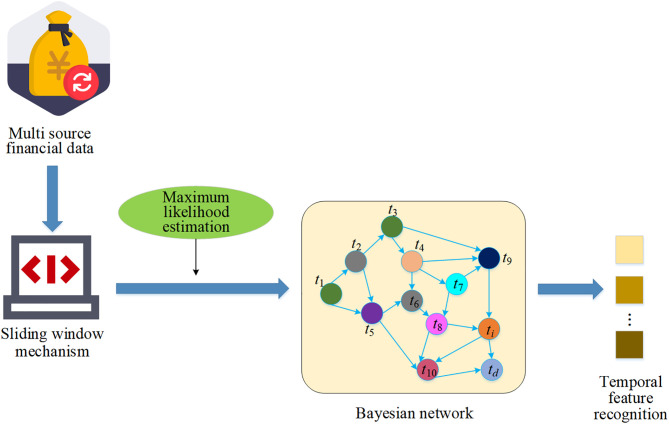
Hierarchical structure in the DBN.

To capture the causal structure and conditional probability responses within multivariate financial time series, this study constructs a hierarchical DBN [31] based on the attention dependency matrix derived from the Transformer with temporally causal masking, as illustrated in Fig 4. This structure supports dynamic modeling of asset price formation mechanisms and high-frequency forecasting.

The DBN operates on a time-slice basis, where each time slice *t* comprises a static Bayesian network $B_{t}\;=\left(G_{t}\;,\theta_{t}\;\right)$. Here, $G_{t}$ represents the structural graph (i.e., a DAG among variables), and $\theta_{t}$ denotes the conditional probability distributions of each node. By incorporating inter-slice temporal edges, a complete time-unrolled network is formed. Let the set of random variables at time *t* be $X_{t}\;=\{\overset{\left(1\right)}{\underset{t}{X}}\;,\overset{\left(2\right)}{\underset{t}{X}}\;,...,\overset{\left(d\right)}{\underset{t}{X}}\;\}$, representing all financial features at that time step. The joint probability distribution of *t*he DBN is expressed as Equation (7):


$$P\left(X_{1:T}\;\right)=P\left(X_{\text{1}}\;\right)\prod_{t=2}^{T}P\left(X_{t}\;|X_{t-1}\;\right)$$ (7)

Within each time slice, conditional independence is defined by the graph structure $G_{t}$. Dependencies between slices are typically assumed to follow a first-order Markov process, where each state depends only on the immediately preceding time step.

To enhance the interpretability of the model and its alignment with financial semantics, this study implements a hierarchical modeling structure within the network. The top layer models the causal transmission pathways from macroeconomic variables (such as interest rates and policy risk factors) to systemic market risk factors. The bottom layer captures the responsiveness of individual stock prices to industry indicators and macroeconomic variables.

This hierarchical structure is formally represented as a conditional probability chain, as shown in Equation (8):


$$P(\overset{\left(i\right)}{\underset{t}{P}}|I_{t}\;,M_{t}\;)=P(\overset{\left(i\right)}{\underset{t}{P}}|I_{t})\cdot P\left(I_{t}\;|M_{t}\;\right)$$ (8)

In Equation (8), $\overset{\left(i\right)}{\underset{t}{P}}$ denotes the price of stock *i* at time *t*, $I_{t}$ represents the industry fac*t*or vector, and $M_{t}$ refers to the macroeconomic factor vector.

To balance model complexity with learning efficiency, this study introduces hierarchical constraints based on domain knowledge, where the top layer simulates macro environments and the bottom layer captures individual stock responses. To address the frequency mismatch between macro indicators and trading data, a “state-context mapping” strategy is employed to align variables by treating macro factors as persistent backgrounds for high-frequency trading. Such structural constraints significantly narrow the search space of the Bayesian network, enabling the model to maintain computational parsimony even when processing high-dimensional features. Meanwhile, real-time features extracted by the Transformer allow the model to dynamically revise its reliance on prior structures, achieving an adaptive balance between expert expertise and data-driven insights.

To extract effective variable dependency structures from attention weights generated by the Transformer, this study employs the average attention matrix *α* as the initial indicator of dependency strength. By setting an empirical threshold *τ*, as shown in Equation (9), the study filters correlation edges with significant structural impact on price fluctuations. Subsequently, acyclicity constraints are applied to generate candidate network topologies for each time slice, ensuring the structural validity of the resulting probabilistic graphical models.


$$E=\{(X_{i}\;,X_{j}\;)\text{|}\alpha_{ij}\;>\tau ,i\neq j\}$$ (9)

In Equation (9), the threshold *τ* is empirically set to 0.05 to retain only those variable dependencies that have a significant influence on price fluctuations.

Subsequently, feature representations produced by the Transformer and those derived from the DBN structure are integrated in a synchronized manner. At each time step, the Transformer encoder outputs a time series embedding $H_{t}\;=\{\overset{\left(1\right)}{\underset{t}{h}}\;,...,\overset{\left(d\right)}{\underset{t}{h}}\;\}$, where each embedding vector $\overset{\left(i\right)}{\underset{t}{h}}$ represents the contextual representation of the financial variable $X^{\left(i\right)}$ at time *t*. Then, the cross-variable attention weight matrix *α*_*t*_ is used as a candidate causal edge strength matrix, which is subjec*t*ed to threshold filtering and acyclicity constraints to generate the candidate network structure $G_{t}$ for time slice *t*.

The overall optimization objective of the model is defined by the following joint loss function, as shown in Equation (10):


$$L_{total}\;=L_{pred}\;+\lambda_{\text{1}}\;\cdot L_{dag}+\lambda_{\text{2}}\;\cdot L_{risk}$$ (10)

In Equation (10), $L_{pred}$ denotes the prediction error term, $L_{dag}$ represents the DAG constraint term, and $L_{risk}$ denotes the risk adjustment term. *λ*_1_ and *λ*_2_ are weighting coefficients for balancing these objectives.

The prediction error term $L_{pred}$ is defined in Equation (11):


$$L_{pred}\;=\frac{\text{1}}{N}\sum_{i=1}^{N}{\left({\hat{y}}_{i}-y_{i}\right)}^{\text{2}}$$ (11)

This term measures the mean squared error between the predicted values (e.g., asset prices) and the actual values, where ${\hat{y}}_{i}$ denotes the model output and $y_{i}$ signifies the ground truth.

The DAG constraint term $L_{dag}$ is defined in Equation (12):


$$L_{dag}=\text{Tr}\left(e^{A⊙A}\right)-d$$ (12)

In Equation (12), $L_{dag}$ enforces acyclicity in the structural graph *A*, ⊙ denotes the Hadamard (element-wise) product, and *d* is the number of nodes. This constraint prevents the formation of loops within the graph structure.

The risk adjustment term $L_{risk}$ is given in Equation (13):


$$L_{risk}=\sum_{t}Var\left({\hat{R}}_{t}\right)-\mu E\left[{\hat{R}}_{t}\right]$$ (13)

To specifically quantify the risk adjustment term, this study introduces a refined approach to risk measurement. First, the predicted return ${\hat{R}}_{t}$ is calculated using a rolling-window estimate of asset returns to capture short-term market volatility. Specifically, the return at time *t* is computed as Equation (14):


$${\hat{R}}_{t}=\frac{P_{t}-P_{t-1}}{P_{t-1}}$$ (14)

In Equation (14), $P_{t}$ is the asset price at time *t*, and $P_{t-1}$ is the asset price at *t*he previous time step.

Next, based on the past *N* time steps within a rolling window, the mean μ and variance$Var\left({\hat{R}}_{t}\right)$ of the returns are calculated as Equations (15), (16):


$$\mu =\frac{\text{1}}{N}\sum_{t-N}^{t}{\hat{R}}_{t}$$ (15)

$$Var\left({\hat{R}}_{t}\right)=\frac{\text{1}}{N}\sum_{t-N}^{t}({\hat{R}}_{t}-\mu {)}^{\text{2}}$$ (16)

On this basis, the risk-aversion coefficient *λ* is set as a parameter linked to market volatility, enabling adaptive adjustment according to different market conditions. The coefficient *λ* dynamically responds to overall market volatility and is positively correlated with the market standard deviation (historical volatility). In this way, the model automatically recalibrates its risk preference according to changing market risk.

Furthermore, the model introduces the Sharpe ratio as a dynamic adjustment factor for risk weighting, automatically increasing risk penalties during severe market volatility or periods of declining returns. This mechanism encourages more cautious prediction results to hedge against downside risks effectively. It should be clarified that the simulated trading results serve as evidence for the economic value of the model. Although risk-aversion preferences are internalized within the loss function, the backtesting environment remains unable to fully encompass all real-world slippage, liquidity shocks, and friction costs. Consequently, the interpretation of simulated profitability requires a cautious evaluation that incorporates specific execution constraints and market depth.

To accommodate the dynamic nature of financial markets, a sliding window mechanism is also designed to enable online updates of both the Bayesian network structure and its conditional probability parameters. Notably, although incorporating causal structure learning increases computational complexity, the sliding window mechanism allows for incremental updates, avoiding repeated training on the full historical dataset and significantly reducing the computational cost of each inference. Combined with modern GPU parallelization, the framework meets the stringent real-time requirements of high-frequency quantitative trading. The update procedure is as follows:

1) At each interval Δt, the network structure $G_{t}$ is refreshed by computing the average of attention matrices within the current window $W_{t}=\left\{t-w,...,t\right\}$, and generating a new dependency graph accordingly.
2) The conditional probability distributions $P\left(X_{j}|Pa_{j}\;\right)$ are updated on the windowed samples using maximum likelihood estimation (MLE).
3) The updated Bayesian network $B_{t}\;=\left(G_{t}\;,\theta_{t}\;\right)$ is synchronized with the Transformer representations and jointly participates in the subsequent prediction training.

At any given time step *t*, given the input sequence $X_{t-k:t}$, the model produces the predicted asset price ${\hat{P}}_{t+1}$ via the following process. The final output is then passed to an output layer or decision module for use in a trading system.

Through this procedure, the model achieves an end-to-end optimization that integrates structure, representation, and parameter learning. This enhancement enables adaptive and interpretable modeling for financial asset pricing in complex high-frequency scenarios.

**Fig 5:**
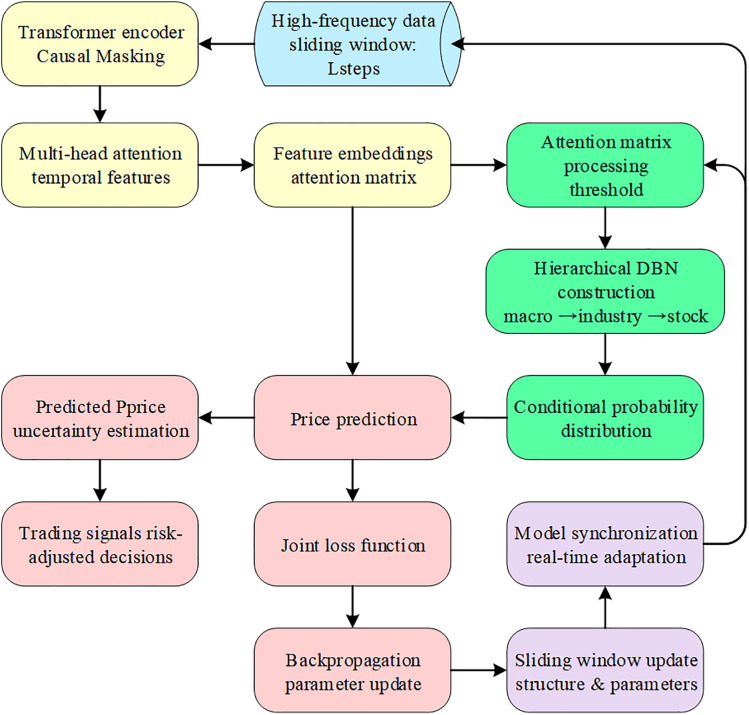
Workflow of the Trans-EFOBN model.

The workflow of the Trans-EFOBN model is illustrated in Fig 5.

**Fig 6:**
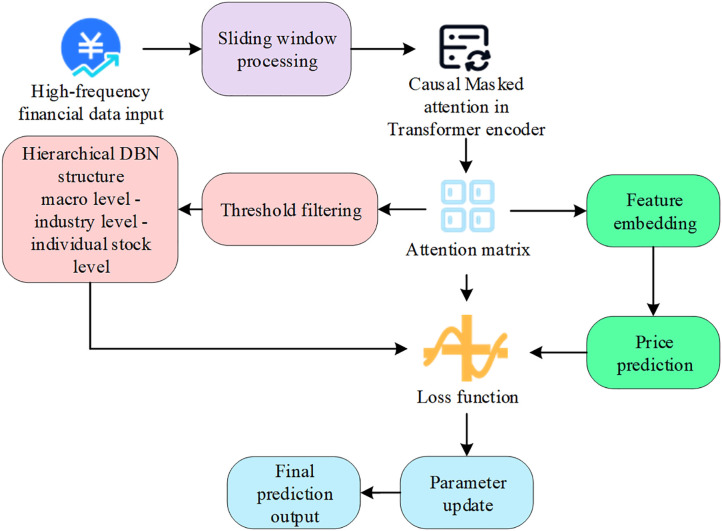
Core technical architecture of the Trans-EFOBN model.

Fig 6 illustrates the comprehensive technical architecture of the Trans-EFOBN model. To enhance clarity while maintaining logical integrity, several complex hyperparameter calculation processes and sub-module details are relocated to the Supporting Information. This figure focuses on the core workflow from feature extraction to structural inference, enabling readers to intuitively understand the conversion of Transformer weights into DBN structural constraints. Consequently, this visualization presents the logical framework of the proposed model with greater precision and coherence.

Fig 6 illustrates the complete technical process of the Trans-EFOBN model from data input to prediction output. It includes the key modules of Transformer feature extraction, attention matrix generation, hierarchical DBN construction, and multi-objective joint optimization. The figure embodies the model’s design philosophy of integrating temporal sequence modeling with causal inference.

**Fig 7:**
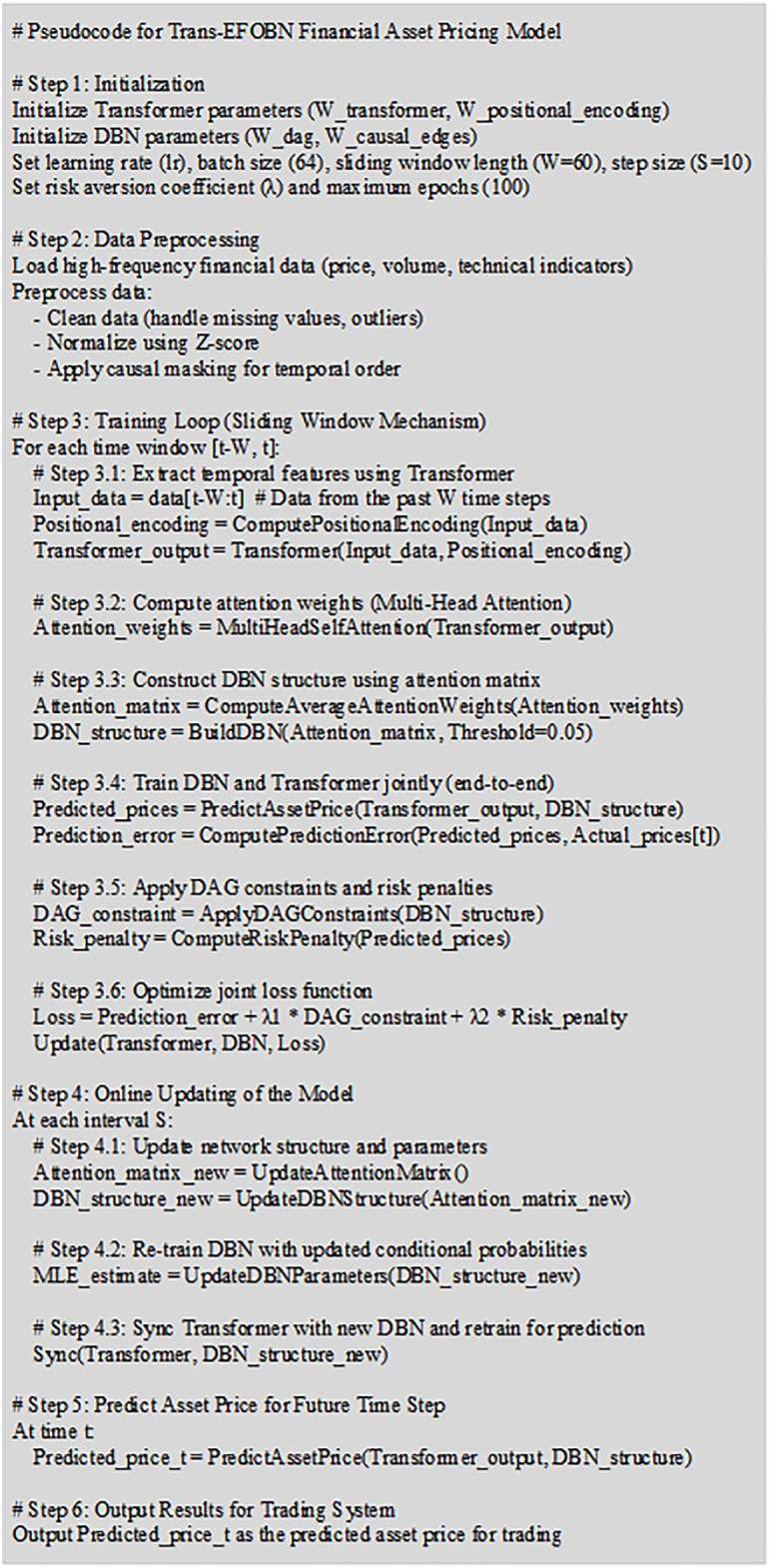
Pseudocode flowchart of the Trans-EFOBN-based financial asset pricing model.

The overall pseudocode for the proposed Trans-EFOBN-based financial asset pricing model is shown in Fig 7.

### Experimental evaluation

To systematically evaluate the efficacy of the Trans-EFOBN model within authentic high-frequency environments, this study constructed a multi-dimensional dataset covering complete bull and bear cycles from 2019 to 2024 based on the Wind database (https://www.wind.com.cn/mobile/WDS/zh.html) [32]. Adhering to principles of high market capitalization, liquidity, and sectoral representation, the study selected several core assets from the CSI 300 index, including China Merchants Bank (600036.SH), Industrial and Commercial Bank of China (601398.SH), China Construction Bank (601939.SH), and Ping An Bank (000001.SZ). During the simulation backtesting process, a 0.1% bilateral transaction cost (including stamp duty and commissions) and a 1 basis point (BP) execution slippage were set. These settings aim to simulate real-world micro-market structure frictions. Although the reduction in mean absolute error (MAE) exhibits marginal effects in numerical terms, the introduction of structured paths significantly enhances the logical stability of the pricing model during extreme volatility. These representative financial stocks possessed exceptional minute-level trading activity, effectively capturing the dynamic impacts of macro policy shocks and micro market interactions, as detailed in Table 2. While this sample represented the high-liquidity Chinese market, the generalizability of these conclusions to international markets or low-liquidity assets required further validation. To ensure reproducibility, this study provided desensitized standardized datasets, preprocessing scripts, and core model code in the Supporting Information, despite copyright restrictions on the original data.

**Table 2:** Experimental Dataset Statistics.

Dimension	Description
Sample period	January 1, 2019 – March 31, 2024 (including COVID-19 and policy adjustment periods)
Asset selection criteria	CSI 300 constituent stocks; daily average turnover > ¥500 million; leading banks/financial institutions
Data frequency	Stock data: minute-level (1 min / 5 min / 30 min) Macro data: quarterly (aligned via forward filling)
Input variables (X)	Open, close, high, low prices; trading volume; VROC, OBV, MACD; macro factors
Total samples	Approx. 240,000 minute-level time steps per stock
Dataset split	Training set: 80%; Test set: 20%
Sliding window	Window length W = 60 minutes; step size = 10 minutes

Data acquisition and preprocessing procedures are designed to ensure high-quality, temporally consistent model inputs. After cleaning, the raw minute-level trading data and quarterly macro indicators are cleaned to remove non-trading periods and holidays, and extreme outliers are smoothed. To address the frequency mismatch between macroeconomic and trading data, the aforementioned “state background mapping” strategy is applied, forward-filling quarterly data to align with the minute-level timeline. All input variables are then standardized using Z-score normalization to eliminate scale differences that could affect gradient descent. To maintain causal consistency, all features involving future information (e.g., lagged technical indicators) are strictly timestamped to prevent data leakage during testing. The final standardized time series samples are split into training (80%) and test (20%) sets, with the split strictly following temporal order to simulate realistic backtesting scenarios.

To comprehensively evaluate the performance of the proposed Trans-EFOBN model, it is compared with several baseline algorithms: Static Bayesian Network (SBN) [33], DBN [34], GNN, Auto-Regressive Transformers (AR-Transformer), Attention-Augmented Transformers (AA-Transformer), Attention-Guided Bayesian Network (Att-BN) [35], and the model proposed by Mishra et al. (2024). Performance metrics include mean squared error (MSE), root mean squared error (RMSE), mean absolute error (MAE), Mean Absolute Percentage Error (MAPE), Mean Absolute Relative Error (MARE), Root Mean Squared Percentage Error (RMSPE), Mean Squared Relative Error (MSRE), Root Mean Squared Relative Error (RMSRE), and the Coefficient of Determination (R²).

The training process is implemented using the PyTorch 2.1 framework, with the Adam optimizer and an end-to-end training strategy. Specifically, the temporal embeddings output by the Transformer encoder are jointly optimized with the causal structural parameters of the DBN. During the data processing stage, the dimensionality and features of the input data vary across different processing steps. The raw dataset is denoted as $X_{\text{raw}}\in {\mathbb{R}}^{N\times T\times F}$, where *N* represents the number of samples, *T* signifies the number of time steps, and *F* denotes the number of features per time step, including open price, close price, trading volume, VROC, OBV, and others. After preprocessing, the data is standardized into a time series format, yielding the processed input $X_{processed}\in {\mathbb{R}}^{N\times T\times F}$. The Transformer model outputs an embedding vector $H_{Transformer}\in {\mathbb{R}}^{N\times T\times d_{model}}$, where *dₘₒ_d_ₑₗ* denotes the Transformer embedding dimension. These vectors are subsequently optimized alongside the DBN’s structural parameters to enable end-to-end predictive training. The model’s hyperparameter settings are summarized in Table 3.

**Table 3:** Hyperparameter configuration.

Hyperparameter	Value	Hyperparameter	Value
Sliding window length *W*	60	Batch size	64
Sliding step	10	Bayesian edge threshold *τ*	0.05
Transformer layers	6	DAG constraint weight *λ*₁	1
Attention heads	4	Risk adjustment weight *λ*₂	0.1
Embedding dimension *dₘₒ_d_ₑₗ*	64	Risk aversion coefficient *γ*	0.5
Learning rate	1e^-4^	Maximum training epochs	100

## Results and discussion

### Performance analysis of model algorithms

**Fig 8:**
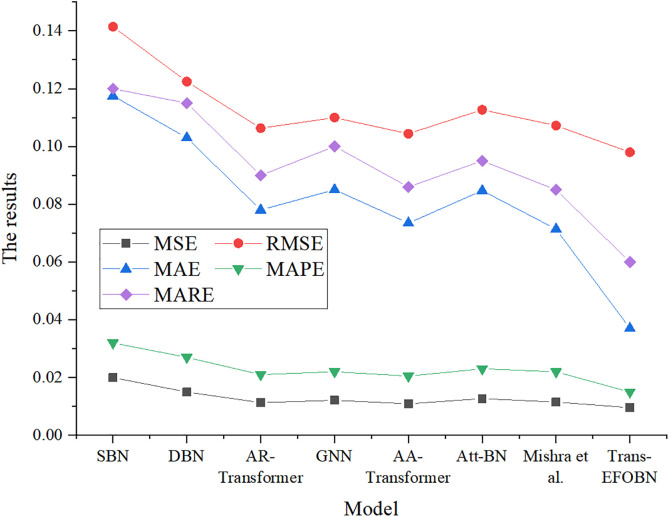
MSE, RMSE, MAE, MAPE, and MARE results for financial asset pricing across different algorithms.

**Fig 9:**
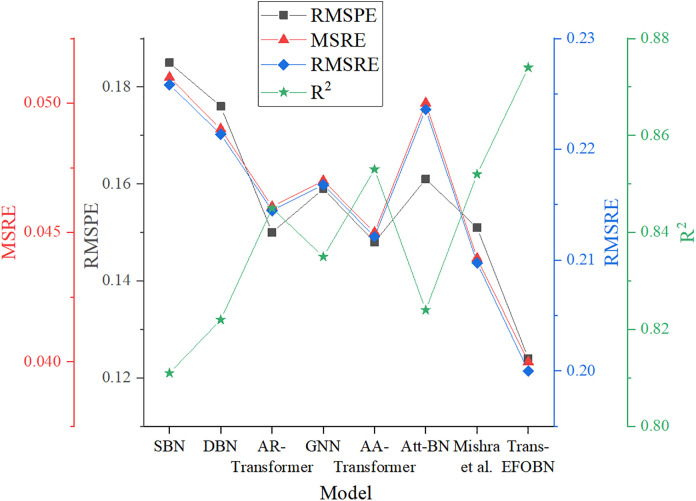
RMSPE, MSRE, RMSRE, and R² results for financial asset pricing across different algorithms.

To evaluate the performance of the proposed Trans-EFOBN model, it is compared with several baseline models, and the results are shown in Figs 8, 9.

As shown in Figs 8,9, the Trans-EFOBN model demonstrates significant advantages across all error metrics. This improvement is not merely statistical; it also reflects the model’s deeper economic value in asset pricing. Specifically, the model reduces MSE and RMSE to 0.0096 and 0.09798, respectively. Compared with SBN and DBN, this notable decrease indicates that the Transformer’s temporal feature extraction effectively captures market irrationality, such as exuberance and panic—i.e., price deviations from fundamentals (“noise”). More importantly, the excellent performance in MAE and MAPE (0.037 and 0.015) highlights the model’s robustness in one-sided market movements. This implies that, in practical trading, the model can more accurately anchor assets to their fair value, thereby reducing execution slippage caused by pricing errors. The high R² value (0.874) achieved by Trans-EFOBN further confirms that the model effectively captures price movements driven by systemic volatility by explicitly modeling structural dependency paths between variables. This design emphasizes enhancing structured representation rather than inferring strict econometric causality, rendering the model more robust than pure association models when facing sudden market shocks. Consequently, the proposed framework provides a solid logical foundation for constructing hedging strategies, ensuring that pricing benchmarks remain reliable even within highly uncertain and volatile financial environments.

To further demonstrate the proposed method’s advantage in capturing complex temporal dynamics, this study conducts a systematic comparison with mainstream time series models, including LSTM, TCN, and Informer. The results are summarized in Table 4. While LSTM can capture basic temporal dependencies, its performance is limited (MAE = 0.055) due to difficulties in handling very long sequences and the lack of explicit structural dependency modeling. TCN improves upon this through dilated convolutions (MAE = 0.048) but remains constrained by its receptive field size. Although Informer leverages sparse attention for efficiency in long sequences (MAE = 0.042), it may overlook the dense, short-term causal fluctuations that are critical in high-frequency trading. In contrast, Trans-EFOBN achieves the best performance across all metrics (MAE = 0.037, R² = 0.874). This advantage stems from the model’s unique integration of the Transformer’s global receptive field with the structured causal reasoning of a DBN. This combination effectively filters noise and captures market factor transmission paths that pure time series models often overlook.

**Table 4:** Performance comparison with mainstream time series models.

Model	MAE	RMSE	MAPE	R^2^
LSTM	0.055	0.138	0.025	0.795
TCN	0.048	0.125	0.021	0.82
Informer	0.042	0.11	0.018	0.845
Trans-EFOBN (Ours)	0.037	0.098	0.015	0.874

When comparing model performance, computational complexity, efficiency, and cost are also critical factors alongside prediction accuracy. Table 5 summarizes these aspects for various models:

**Table 5:** Comparison of computational complexity, efficiency, and cost across models.

Model	Computational Complexity	Time per 1000 Samples	Efficiency	Computational Cost
SBN	O(n²)	0.1 s	Low	Low (CPU)
DBN	O(n²)	0.2 s	Medium	Medium (CPU)
GNN	O(n³)	5.0 s	Low–Medium	High (GPU)
AR-Transformer	O(n² * d)	4.5 s	High	High (GPU)
AA-Transformer	O(n² * d)	4.8 s	High	High (GPU)
Att-BN	O(n²)	0.15 s	Medium	Medium (CPU/GPU)
Mishra et al.	O(n²)	0.2 s	Medium	Medium (CPU/GPU)
Trans-EFOBN	O(n² * d + n³)	7.0 s	High	Very High (GPU)

As shown in Table 5, the models differ significantly in computational complexity, efficiency, and cost. SBN and DBN exhibit a complexity of O(n²) with short computation times (0.1 s and 0.2 s, respectively) and low efficiency and costs, making them suitable for small-scale data processing. GNN requires a complexity of O(n³) and a computation time of 5 s; its medium efficiency and high cost limited its use to handling complex network structures. The AR-Transformer and AA-Transformer featurea complexity of O(n² * d), with computation times of 4.5 s and 4.8 s, respectively. They achieve high efficiency but rely heavily on GPUs, resulting in higher operational costs. Att-BN and the model by Mishra et al. demonstrate O(n²) complexity with moderate computation time, suitable for medium-scale data processing. Although the computational complexity of the Trans-EFOBN model reaches $O(n^{2}\cdot d+n^{3})$, which increases the computational cost compared to simple Static Bayesian Network (SBN), this complexity is in exchange for a deep perspective on the price formation mechanism. Through SHAPley Additive exPlanations (SHAP) value analysis, it is possible to clearly identify the contribution weights of historical prices and macro variables at different frequencies. This transparency provides key support for risk prevention and control in high-frequency trading.

Although Table 5 reveals higher computational costs for Trans-EFOBN (7.0 seconds per thousand samples), this remains feasible for practical quantitative trading deployment. The model adopts an “offline training, online inference” architecture, where complex structural learning and joint optimization are completed offline, while online prediction involves only matrix forward operations. This design fully satisfies the time constraints of minute-level trading and can further reduce latency through GPU parallel computing and distributed optimization. For institutional investors pursuing high-frequency responses, exchanging moderate computational resources for superior pricing precision and logical transparency yields significant practical benefits.

### Model sensitivity and feature importance analysis

**Fig 10:**
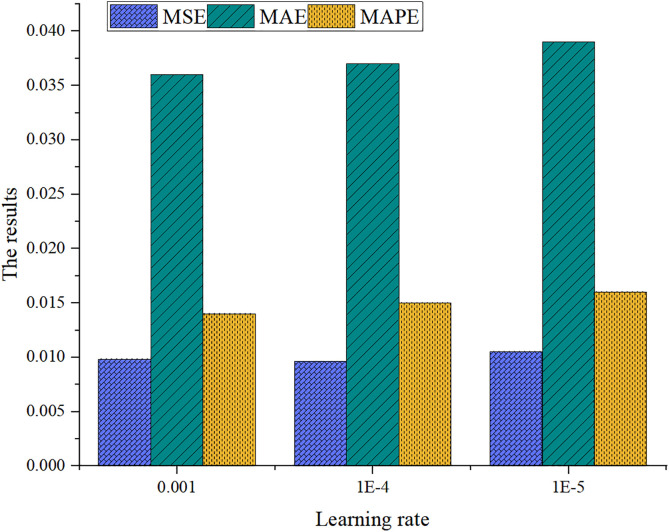
Learning rate sensitivity analysis.

To assess the sensitivity of the Trans-EFOBN model, this study varies the learning rate [0.001, 1e-4, 1e-5] and the number of Transformer layers [4,6,8]. Model performance under these configurations is evaluated using MAE, MSE, and MAPE metrics. Fig 10 displays the impact of different learning rates on model performance for a six-layer Transformer configuration.

**Fig 11:**
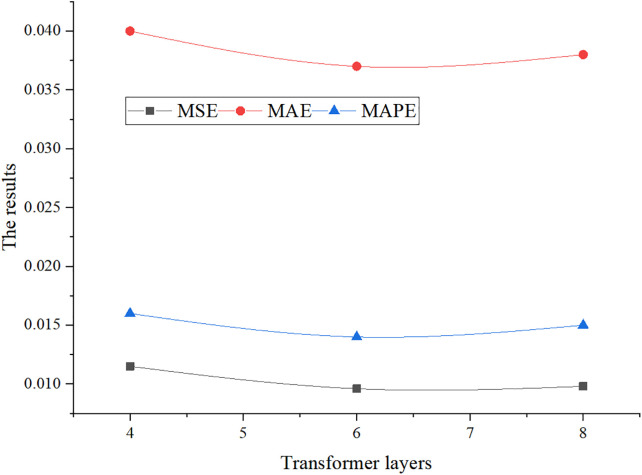
Transformer layer sensitivity analysis.

Fig 11 illustrates the effect of varying the number of Transformer layers on performance when the learning rate is fixed at 0.0001.

From Figs 10, 11, it is evident that the model achieves optimal performance in terms of MSE, MAE, and MAPE when the learning rate is 1e-4 and the Transformer contains six layers. Increasing the number of layers does not yield significant improvements. Conversely, learning rates of 1e-3 and 1e-5 lead to performance degradation, particularly in MAE and MAPE. Therefore, a moderate Transformer depth (6 layers) combined with a relatively low learning rate (1e-4) optimizes predictive performance, whereas extreme learning rates may cause overfitting or slow convergence.

SHAP values provide an effective method for assessing feature importance by quantifying the contribution of each feature to the model’s predictions, thereby enhancing interpretability. In the Trans-EFOBN model, SHAP values reveal how different financial features—such as historical prices, trading volume, and technical indicators—impact asset price predictions. By calculating these contributions, the most influential features are identified. Table 6 presents the SHAP value contributions of various features in the Trans-EFOBN model.

**Table 6:** SHAP value contributions of features in the Trans-EFOBN model.

Feature	SHAP Contribution (%)	Feature	SHAP Contribution (%)
Historical Price	35.2%	Moving Average Convergence Divergence (MACD)	6.3%
Trading Volume	18.4%	Exponential Moving Average	5.1%
Moving Average	12.8%	Market Sentiment Index	4.1%
Relative Strength Index	8.5%	Gross Domestic Product (GDP) Growth Rate	1.7%
Bollinger Bands	7.9%	Inflation Rate	1.4%

The feature distribution revealed in Table 6 provides a logical basis for understanding high-frequency pricing mechanisms. The significant contributions of historical prices (35.2%) and trading volume (18.4%) align with market common sense regarding volume-price relationships driving short-term fluctuations. Notably, macro variables like GDP and inflation show low direct contributions (<2%) in minute-level predictions, which does not imply their insignificance within the framework. In the Trans-EFOBN architecture, macro factors primarily function as “state contexts” that implicitly shape risk preferences through the hierarchical structure of the Bayesian network. This hierarchical attribution demonstrates that the model effectively distinguishes between long-cycle fundamentals and short-cycle emotional features, achieving more reasonable logical explanations than pure time-series models.

### Ablation study

**Fig 12:**
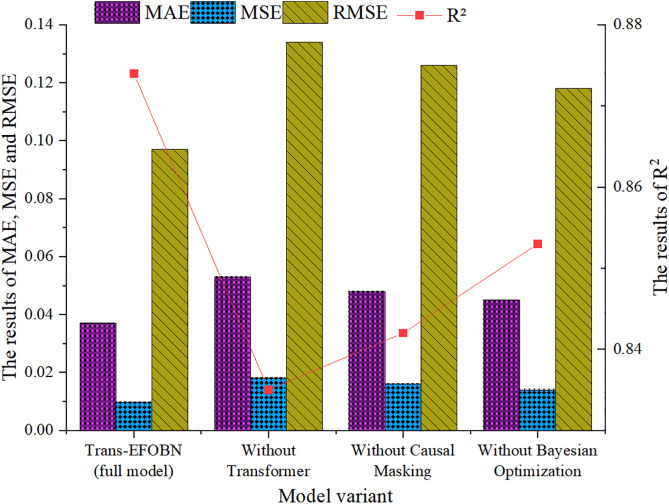
Ablation study results for the Trans-EFOBN model.

To comprehensively understand the contribution of each component in the Trans-EFOBN model, an ablation study is conducted. The Transformer module, temporally causal masking mechanism, and Bayesian optimization component are individually removed to quantitatively assess their impact on model performance. The results are illustrated in Fig 12.

The results show that the full Trans-EFOBN model outperforms any variant with a removed component. When the Transformer module is removed, MAE rises to 0.053, MSE to 0.018, RMSE to 0.134, and R² drops to 0.835, compared with the full model’s MAE of 0.037 and R² of 0.874. This demonstrates that the Transformer is crucial for capturing long-range dependencies and complex nonlinear relationships in time series data. Removing the temporally causal masking mechanism also reduces performance, with MAE increasing from 0.037 to 0.048. This highlights its role in modeling causal dependencies in financial data. Experiments demonstrate that Trans-EFOBN outperforms any single-component variant, indicating that the integration of Transformer and Bayesian modules yields superior predictive results. Notably, although Bayesian optimization increases model complexity, the resulting MAE reduction from 0.041 to 0.037 carries significant economic marginal value in high-frequency pricing scenarios. While the Transformer captures long-range nonlinear features, the Bayesian structure filters temporal noise by imposing logical constraints on the variable dependency paths. This synergistic mechanism of “feature extraction plus logical inference” proves the necessity of the hybrid architecture for enhancing both precision and transparency, rather than simple redundancy.

### Performance analysis under different temporal granularities

**Fig 13:**
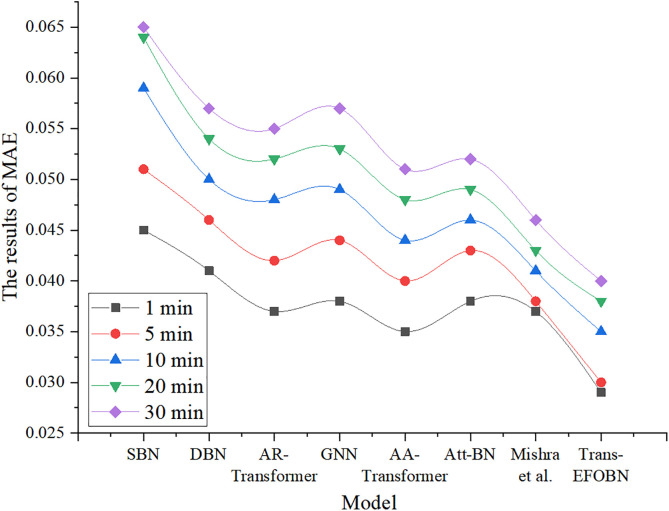
MAE results of different algorithms under varying temporal granularities.

To evaluate model performance across varying temporal granularities, the MAE results for financial asset pricing across different algorithms are presented in Fig 13.

As shown in Fig 13, the Trans-EFOBN model achieves the lowest MAE across different time granularities, outperforming all other models particularly at the 1-minute and 5-minute levels. Specifically, at the 1-minute granularity, Trans-EFOBN attains an MAE of 0.029, significantly better than baseline models such as SBN (0.045) and DBN (0.041), and even lower than the model by Mishra et al. (0.037). At longer time granularities, such as 30 minutes, Trans-EFOBN maintains a low MAE of 0.04, demonstrating its robustness and superior performance across time scales. In comparison, while models like GNN, AR-Transformer, and AA-Transformer perform well at specific granularities, they do not match the overall consistency of Trans-EFOBN. These results indicate that Trans-EFOBN possesses strong generalization ability across multiple time granularities, providing more accurate predictions for financial asset pricing in high-frequency environments.

### Economic impact analysis

**Fig 14:**
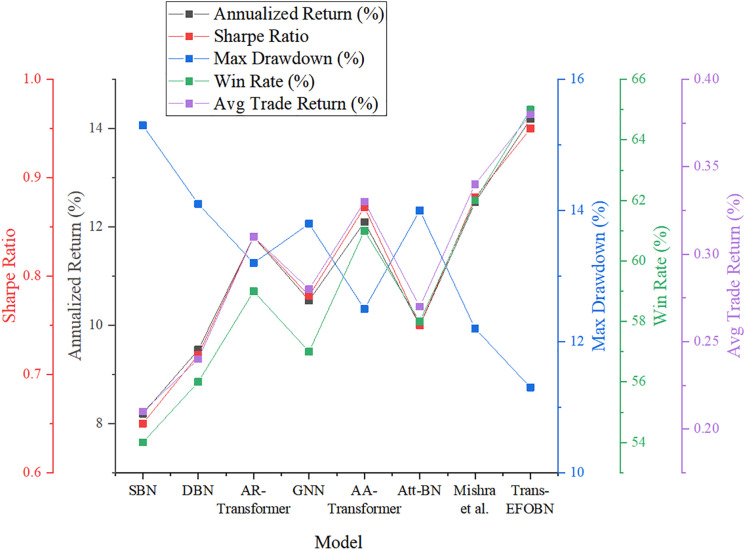
Economic performance analysis of different models.

Fig 14 compares the economic performance of the Trans-EFOBN model against baseline models in simulated trading scenarios.

As shown in Fig 14, the simulated trading results indicate that Trans-EFOBN delivers both statistically significant performance and substantial economic value. Its annualized return of 14.2% and Sharpe ratio of 0.95 demonstrate that the model, through the DBN, successfully identifies mispricing opportunities in the market while effectively mitigating idiosyncratic risk. In simulated trading evaluations, Trans-EFOBN demonstrates strong drawdown resistance, maintaining stable performance even during significant market fluctuations. To align the economic impact analysis with reality, backtesting incorporates a 0.1% bilateral transaction cost (including commissions and stamp duty) and a 1-basis-point slippage assumption. Despite these adjustments, real-time liquidity constraints and market shocks during extreme conditions may still influence actual profitability within live trading environments. Future research will further integrate more sophisticated execution algorithm simulations to verify the robustness of the model after accounting for all realistic friction costs.

### Discussion

The proposed Trans-EFOBN framework not only surpasses existing benchmarks in predictive accuracy but also offers a novel theoretical perspective for understanding the mechanism through which information flows into prices in high-frequency markets. Unlike Song et al. (2024) [36], who focused on risk monitoring using Transformer and LSTM models, the proposed model employs a temporally causal masking mechanism that goes beyond capturing sequential correlations. This design decouples the structural drivers underlying market volatility, offering a clearer view of price formation. Furthermore, this architecture addresses the challenge highlighted by Kim et al. (2023) [37] regarding S&P 500 index prediction using FinBERT and LSTM. Pure deep learning models often failed to explain why specific sentiment signals led to markedly different price reactions under varying market conditions. In the Trans-EFOBN framework, the hierarchical Bayesian structure functions as a “logical interpreter.” It reveals how macroeconomic conditions act as conditional variables that dynamically modulate the influence of micro-level sentiment on asset pricing.

Regarding causality-driven prediction paradigms, Díaz Berenguer et al. (2024) [38] recently emphasized the necessity of incorporating structural dependencies in multivariate stock forecasting. Compared with their approach, Trans-EFOBN offers the advantage of end-to-end joint optimization of feature extraction and structural learning. While the structural dependencies in this study are inferred through the model’s hierarchical structure and attention-based dependency reasoning rather than formal econometric causal identification, this design effectively leverages Transformer attention weights to guide the real-time evolution of the Bayesian network structure. This enables the model to adapt to the “Adaptive Market Hypothesis”, where causal dependencies rapidly switch between fundamentals-driven and sentiment-driven regimes.

Finally, from the perspective of algorithmic robustness, this study introduces a risk-adjusted loss function that internalizes the risk-aversion preferences of Modern Portfolio Theory at the algorithmic level. This design overcomes the limitations of pure predictive models, such as those of Song et al. [21] and Kim et al. [37], which overlooked tail risks during extreme market conditions. Empirical results show that the proposed hybrid modeling strategy effectively balances predictive accuracy—by minimizing MSE—with structural rationality—by maximizing model transparency / structural logic. This combination is a key pathway to improving the resilience of quantitative trading strategies in complex and dynamic market environments.

## Conclusion

The proposed Trans-EFOBN model significantly enhances financial asset pricing efficiency by integrating frequency enhancement with structured modeling techniques. Empirical results demonstrate that the model achieves an R² of 0.874 on CSI 300 constituent stocks, outperforming traditional Bayesian networks and advanced deep learning models in error metrics like MAE. The core advantage of Trans-EFOBN lies in balancing predictive precision with structural transparency, utilizing Transformers to capture nonlinear features while reducing “black-box” uncertainty through hierarchical logical structures. Economic evaluations further confirm its robustness within complex high-frequency scenarios, providing reliable pricing benchmarks under volatile market conditions. Although higher model complexity increases computational costs, the gains in risk identification and logical interpretability offer essential technical references for developing intelligent quantitative trading strategies.

Moreover, the findings of this study hold significant implications for financial regulation and policymaking. Empirical results indicate that explicitly incorporating macroeconomic states into high-frequency pricing models significantly improves pricing efficiency. This suggests that the transparency of monetary policy and macroprudential regulatory information is crucial for stabilizing micro-market structures. For regulators, this implies that increasing the temporal granularity of policy announcements or establishing real-time market communication mechanisms could reduce information asymmetry and speculative noise in high-frequency trading, thereby guiding capital flows toward the fundamentals. Additionally, the model’s sensitivity to extreme volatility provides micro-level data support and theoretical guidance for exchanges in designing more effective circuit breakers and volatility control measures.

Despite its strengths, the Trans-EFOBN model possesses several limitations that provide pathways for future enhancement. Its structure optimization process requires learning complex structural dependencies, which leads to high computational costs. The current empirical scope mainly focuses on the Chinese A-share market with abundant liquidity, which to some extent limits the generalizability of the conclusions. The regulatory environment and market microstructure in different jurisdictions may lead to a drift in the strength of structural dependence between variables. Future exploration will focus on migrating the framework to different asset classes such as commodity futures or forex to validate its robustness in heterogeneous data environments. Meantime, in response to the frequency mismatch between macro indicators such as Gross Domestic Product (GDP) and high-frequency trading data, it is planned to introduce higher frequency real-time forecast data to enhance synchronization.

## Supporting information

S1 Appendix Glossary of Terms. A list of key technical terms and their definitions used in this manuscript.
(DOCX)

S2 Code The code of the core algorithm section of the manuscript. (TXT)

S3 Data Figures data from Fig 8–Fig 14. (XLSX)

S4 File This model description helps other reader understand the core code. (DOCX)
