# Supplementary material for: Enhancing the pricing efficiency of financial assets with an optimized bayesian network based on efficient fusion
Source: PLoS One. 2026 May 8;21(5):e0347047. doi: 10.1371/journal.pone.0347047 (PMC13155572; doi:10.1371/journal.pone.0347047)
Supplement: S1 Appendix — A list of key technical terms and their definitions used in this manuscript. (DOCX) [file pone.0347047.s001.docx]

**Appendix**

**Glossary of Terms**

| Category | Belongs to | Belongs to | Description |
| --- | --- | --- | --- |
| Parameters | Input sequence | 𝑋 | Input sequence of financial variables spanning 𝑙 consecutive time steps |
|  | Time step index | 𝑡 | Index of the time step |
|  | Number of financial variables | 𝑑 | Number of financial variables at each time step |
|  | Sliding window length | 𝑊 | Length of the sliding window |
|  | Effective edge threshold | 𝜏 | Threshold for selecting effective edges in the Bayesian network |
|  | Loss balance weights | 𝜆₁, 𝜆₂ | Weights for balancing prediction error and DAG constraints |
|  | Risk aversion coefficient | 𝛾 | Coefficient of risk aversion in the risk adjustment term |
| Abbreviations | Transformer-enhanced Efficient Fusion Optimized Bayesian Network | Trans-EFOBN | Proposed model in this study |
|  | Dynamic Bayesian Network | DBN | Dynamic Bayesian Network |
|  | Directed Acyclic Graph | DAG | Directed Acyclic Graph |
|  | Mean Absolute Error | MAE | Mean Absolute Error |
|  | Mean Squared Error | MSE | Mean Squared Error |
|  | Root Mean Squared Error | RMSE | Root Mean Squared Error |
|  | Mean Absolute Percentage Error | MAPE | Mean Absolute Percentage Error |
|  | Mean Absolute Relative Error | MARE | Mean Absolute Relative Error |
|  | Root Mean Squared Percentage Error | RMSPE | Root Mean Squared Percentage Error |
|  | Mean Squared Relative Error | MSRE | Mean Squared Relative Error |
|  | Root Mean Squared Relative Error | RMSRE | Root Mean Squared Relative Error |
|  | Coefficient of Determination | R² | Coefficient of Determination |
|  | Volume Rate of Change | VROC | Volume Rate of Change |
|  | On-Balance Volume | OBV | On-Balance Volume |
|  | Static Bayesian Network | SBN | Static Bayesian Network |
|  | Attention-Guided Bayesian Network | Att-BN | Attention-Guided Bayesian Network |
|  | Graph Neural Networks | GNN | Graph Neural Networks |
|  | Auto-Regressive Transformers | AR-Transformer | Auto-Regressive Transformers |
|  | Attention-Augmented Transformers | AA-Transformer | Attention-Augmented Transformers |
| Indices | Individual stock index | 𝑖 | Individual stock index |
|  | Financial variable index | 𝑗 | Index of financial variables in the input sequence |
|  | Attention head index | ℎ | Index of an attention head in the multi-head attention mechanism |
|  | Transformer layer index | 𝑙 | Index of a layer in the Transformer model |
|  | DBN time slice index | 𝑡 | Index of a time slice in the DBN |
