# Supplementary material for: Enhancing the pricing efficiency of financial assets with an optimized bayesian network based on efficient fusion
Source: PLoS One. 2026 May 8;21(5):e0347047. doi: 10.1371/journal.pone.0347047 (PMC13155572; doi:10.1371/journal.pone.0347047)
Supplement: S4 File — (DOCX) [file pone.0347047.s004.docx]

**Model code description:**

**1. Causal Mask Transformer**

• Function: Receive multivariate financial sequences (such as prices, trading volumes, technical indicators) within a sliding window, and extract time-dependent features and latent causal representations through a causal masked Transformer encoder.

• Core mechanism: Use positional encoding to preserve temporal order, aggregate cross-temporal features through multi head attention, and introduce masks to ensure that future information is not used, in accordance with causality.

**2. Dynamic Bayesian Network**

• Function: Construct a hierarchical dynamic Bayesian network based on the attention matrix of the Transformer to explore the causal structure and conditional probability response between variables.

• Structural update:

- - Use the average value of the attention matrix for causal edge filtering (set threshold).
  - Ensure that the generated network is a directed acyclic graph (DAG) through acyclic constraints.

• Parameter update:

- - Use maximum likelihood estimation (MLE) to estimate the conditional probability distribution of each edge on the current sliding window data.

**3. Joint optimization and online update process**

• Function: Integrate the structure/parameters of Transformer and Bayesian network into a joint optimization framework, and use a joint loss function for end-to-end training.

• The joint loss function consists of three parts:

- - Prediction Error (MSE)
  - DAG structure constraints (used to prevent Bayesian graphs from forming loops)
  - Risk adjustment item (introducing volatility control to enhance robustness)

**4. train_trans_efobn main function**

• Use sliding windows to gradually process data streams.

• Update the Bayesian network structure/parameters every fixed time step to enhance model adaptability.

• Output the final prediction to the trading system interface (which can be connected to the strategy or order module).

**Hyperparameter configuration instructions:**

| **Hyperparameter** | **Default value** | **Meaning and function** |
| --- | --- | --- |
| WINDOW_SIZE | 60 | Sliding window length: the number of data time steps used for training and structural updates each time. |
| UPDATE_INTERVAL | 10 | Bayesian network structure and parameter update frequency (updated every 10 steps). |
| TAU | 0.05 | Causal edge filtering threshold: edges below this attention weight will be discarded. |
| LAMBDA_DAG | 1 | The weight coefficients of DAG regularization terms, used to prevent the occurrence of closed loops in graph structures. |
| LAMBDA_RISK | 0.1 | The weight of risk penalty items, adjusting the model's emphasis on risk control. |
| RISK_AVERSION | 0.5 | The risk aversion coefficient of investors, which determines the degree of trade-off between returns and volatility. |
| model_dim | 64 | Dimension of embedding vectors in Transformer encoder. |
| input_dim | 10 | Input feature dimensions, representing the number of financial variables included in each time step. |
